# High Rates of Enteric Fever Diagnosis and Lower Burden of Culture-Confirmed Disease in Peri-urban and Rural Nepal

**DOI:** 10.1093/infdis/jix221

**Published:** 2017-07-28

**Authors:** Jason R Andrews, Krista Vaidya, Caryn Bern, Dipesh Tamrakar, Shawn Wen, Surendra Madhup, Rajeev Shrestha, Biraj Karmacharya, Bibush Amatya, Rajendra Koju, Shiva Raj Adhikari, Elizabeth Hohmann, Edward T Ryan, Isaac I Bogoch

**Affiliations:** 1 Department of Medicine, Stanford University, Stanford; 2 Department of Epidemiology and Biostatistics, University of California, San Francisco, California; 3 D-Lab, Massachusetts Institute of Technology, Cambridge; 4 Division of Infectious Diseases, Massachusetts General Hospital, Boston, Massachusetts; 5 Dhulikhel Hospital, Kathmandu University Hospital, Dhulikhel; 6 Department of Economics, Tribhuvan University, Kathmandu, Nepal; 7 Department of Medicine, University of Toronto, Canada

**Keywords:** typhoid, enteric fever, acute febrile illness, Salmonella, epidemiology, Nepal

## Abstract

**Background:**

In South Asia, data on enteric fever are sparse outside of urban areas. We characterized enteric fever diagnosis patterns and the burden of culture-confirmed cases in peri-urban and rural Nepal.

**Methods:**

We used national reports to estimate enteric fever diagnosis rates over 20 years (1994–2014) and conducted a prospective study of patients presenting with a >72-hour history of fever to 4 peri-urban and rural healthcare facilities (during August 2013–June 2016). We compared clinical characteristics of patients with culture-confirmed *Salmonella* Typhi or Paratyphi infection to those of patients without enteric fever. We used generalized additive models with logistic link functions to evaluate associations of age and population density with culture positivity.

**Results:**

National rates of enteric fever diagnosis were high, reaching 18.8 cases per 1000 during 2009–2014. We enrolled 4309 participants with acute febrile illness. Among those with a provisional clinical diagnosis, 55% (1334 of 2412) received a diagnosis of enteric fever; however, only 4.1% of these had culture-confirmed typhoidal *Salmonella* infection. Culture positivity was highest among young adults and was strongly associated with higher population density (*P* < .001).

**Conclusions:**

Enteric fever diagnosis rates were very high throughout Nepal, but in rural settings, few patients had culture-confirmed disease. Expanded surveillance may inform local enteric fever treatment and prevention strategies.

Enteric fever, caused by *Salmonella* Typhi or *Salmonella* Paratyphi, is an important cause of morbidity and mortality globally [1, 2]. More than 10 million cases and 100000 deaths annually are estimated to occur because of these diseases [3–6]. However, estimates vary widely, owing to limited geographical and temporal resolution of the underlying data. Most incidence data are from studies in urban areas, which are often targeted for vaccine trials because of high reported case numbers. In South Asia, regional estimates were derived from just 4 large cities: New Delhi, Kolkata, Dhaka, and Karachi [5]. Whether such data can be generalized to peri-urban or rural populations is unknown.

The need for broader data on the burden of enteric fever is critical to inform treatment and prevention approaches. Antimicrobial resistance to first-line agents, including fluoroquinolones, has become increasingly common and may be further accelerated by widespread use of antibiotics for empirical treatment of nontyphoid febrile illnesses [7, 8]. Fortunately, several conjugate vaccine candidates have demonstrated promise in clinical trials [9]. Where and how such vaccines should be deployed will be a challenging question facing policy makers, funders, and technical agencies in the coming years and will require better understanding of the populations at risk.

In Nepal, nearly all data on enteric fever have been generated within the Kathmandu Valley [10–13], while 90% of the population lives outside this region, predominantly in rural areas [14]. While there have been no systematic data regarding diagnosis and treatment practices for enteric fever in these areas, anecdotal evidence suggests that clinicians report treating enteric fever commonly. How commonly enteric fever is diagnosed, how this varies across the country, and whether diagnosed cases outside of urban areas reflect true disease are not known. We therefore sought to characterize diagnosis and treatment practices and the true burden of enteric fever among patients with acute febrile illnesses in peri-urban and rural parts of the country.

## METHODS

### Ethics Statement

Approval was obtained from the Institutional Review Board for Human Subjects Research of the Nepal Health Research Council (Kathmandu, Nepal), the Kathmandu University School of Medical Sciences Institutional Review Committee (Dhulikhel, Nepal), Partners Human Research Committee (Boston, MA), the University Health Network Research Ethics Board (Toronto, Canada), and the Stanford University Institutional Review Board (Stanford, CA). Eligible participants aged ≥18 years were required to provide written informed consent. For children aged 5–17 years, verbal assent was obtained from the child. For all children, parents or guardians provided written informed consent.

### Overview

This study consisted of 2 components: (1) a secondary analysis of enteric fever notifications by district and year from national healthcare reporting data and (2) a prospective study assessing the enteric fever burden among individuals presenting to health facilities with acute febrile illnesses.

### Secondary Data Analysis

We extracted outpatient department visits and typhoid/paratyphoid diagnoses from annual reports of the Department of Health Services, which collates data from the Health Management Information System (HMIS). The HMIS has been implemented throughout country since 1994 and covers all health services provided through government health facilities, as well as many nongovernment health facilities. Of note, each Department of Health Services report is split across years to coincide with the calendar and reporting year for the Government of Nepal (eg, 2009–2010). We obtained district-wide population estimates from these reports, which were derived from census data from the Central Bureau of Statistics. We calculated the proportion of all outpatient department visits for which typhoid/paratyphoid fever was listed as a diagnosis. We report notification rates as cases reported per 1000 population. We calculated population notification rates for each district by dividing total notifications through the HMIS by the district population and averaged these rates across 5 years (2009–2014).

### Prospective Study Setting and Population

**Figure 1.**
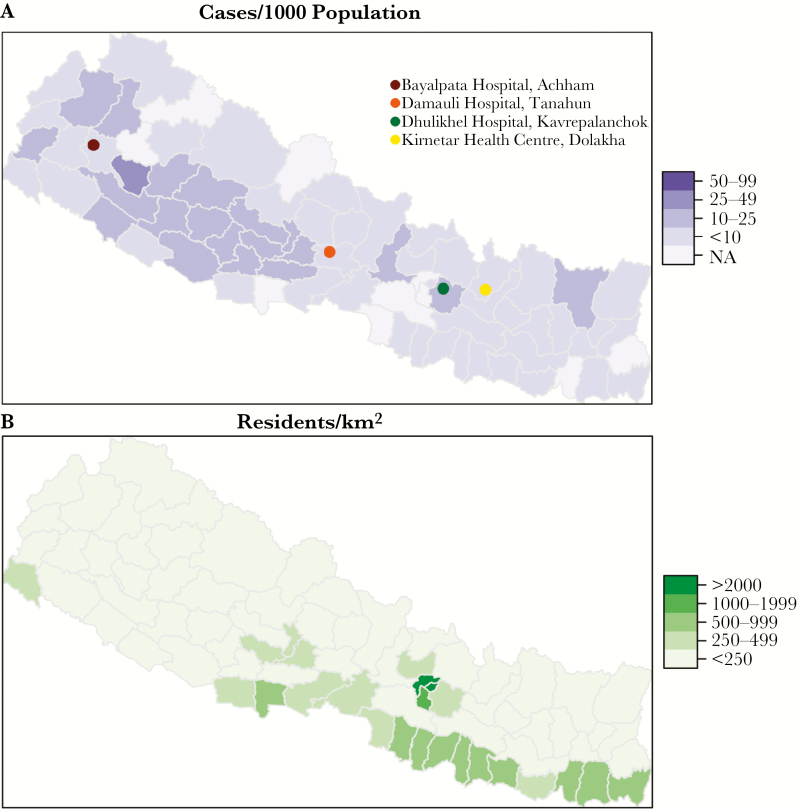
*A*, Typhoid/paratyphoid notifications, by district, during 2009–2014. *B*, Population density, by district. Abbreviation: NA, not available.

The study was performed between August, 2013 and June, 2016 at 4 study sites: Bayalpata Hospital (Achham), Damauli Hospital (Tanahun), Dhulikhel Hospital (Kavrepalanchowk), and Kirnetar Health Centre (Dolakha; Figure 1). Bayalpata Hospital is a 15-bed hospital serving rural villages in an impoverished, hilly district of far-western Nepal. Damauli Hospital is a district hospital serving a town (Vyas) with a population of around 43000. Dhulikhel Hospital is a tertiary-care teaching hospital of Kathmandu University. Located 30 km from Kathmandu, the hospital serves a predominantly peri-urban area but also has a large rural catchment area of 2.5 million. Kirnetar Health Center is a clinic in Dolakha, a rural district east of Kathmandu.

All individuals >12 months of age presenting to the study sites with a self-reported >72-hour history of fever were invited to participate in the study. Participants were recruited from the outpatient departments, emergency department, and inpatient wards.

### Study Procedures

A questionnaire was administered to each participant or adult guardian by a study staff member to ascertain demographic characteristics, including residence locality, symptoms, history of present illness, prior care-seeking behavior, and self- reported antibiotic use during this febrile episode. Population densities for village development committees or municipalities were derived from the 2011 national census population and geographic area data. Residence locality data were not available for the Bayalpata Hospital and Damauli Hospital sites (7% of patients). Additionally, study staff recorded documentation by clinicians, including clinical diagnoses at initial evaluation. A venous blood specimen was collected from all participants (2–5 mL from children aged 1–5 years and 10 mL from participants aged >5 years). Blood samples were inoculated into culture bottles (1–3 mL for children aged 1–5 years and 8–10 mL for participants aged >5 years). Most cultures were collected in Bactec Plus Aerobic/F or Peds Plus/F bottles, while 1 site used glass bottles with locally prepared tryptic soy broth. Blood cultures were incubated for at least 7 days; blind subcultures were then performed. Bacterial identification was performed using standard biochemical tests, including TSI slant, SIM medium, citrate, and urea testing. Antimicrobial susceptibility was evaluated using disk diffusion diameter measurements on Mueller-Hinton agar, following standard methods [15].

### Statistical Analyses

The primary outcome of the prospective study was the proportion of enrolled participants from whom *S.* Typhi or *S*. Paratyphi was isolated in blood culture; these cases were considered culture-confirmed enteric fever. Additionally, we compared culture-confirmed enteric fever proportions according to demographic and clinical characteristics, using a 2-sample test for equivalence of proportions. To evaluate the nonlinear relationship between *Salmonella* culture positivity and age or between culture positivity and population density, we used generalized additive models with logistic link functions and penalized splines. All analyses were performed using R [16].

## RESULTS

### Enteric Fever Diagnoses in National Notification Data

**Figure 2.**
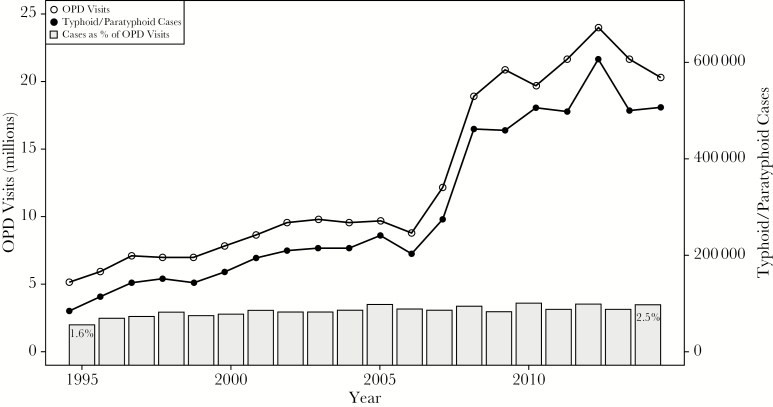
Outpatient department (OPD) visits (open circles; left axis), typhoid/paratyphoid cases reported (solid circles; right axis), and proportion of visits in which typhoid/paratyphoid was reported (gray bars) through the Health Management Information System in Nepal, 1994–1995 through 2013–2014.

Between 1994 and 2014, the overall number of outpatient department visits reported through the HMIS rose from 5.17 million to 20.33 million, with the greatest increase occurring around 2008 upon institution of the national free healthcare policy. The number of typhoid/paratyphoid cases reported rose in parallel, from 85137 to 506563, and proportionally increased from 1.6% to 2.5% of all diagnoses in the outpatient department (Figure 2). During 2009–2014, the annual rate of diagnosis reached 18.8 cases per 1000 population. This rate varied by district, from 1.7 to 52.5 cases, with many of the highest diagnosis rates occurring in rural midwestern and far-western districts (Figure 1).

### Prospective Surveillance for Enteric Fever

From August 2013 through June 2016, 4309 participants were enrolled in 4 study sites. The majority (3529 [81.9%]) were enrolled in Dhulikhel Hospital. Over half of all participants were women (2209 [53.1%]), and the median age was 27 years (interquartile range [IQR], 15–48 years). The median duration of fever prior to presentation was 5 days (IQR, 3–7 days), and only 26.4% were febrile (temperature, >38.3°C [>101°F]) at the time of evaluation. More than half (2367 of 4230 [56.0%]) had sought prior medical care, primarily at pharmacies (771 of 2367 [33%]) and private medical clinics (623 of 2367 [26%]). More than a third of patients (1476 of 4190 [35.2%]) reported antibiotic use prior to presenting to the study site. At the initial study encounter, an empirical diagnosis was made for 2412 patients (56.0%); the most common diagnoses were enteric fever (for 1334 patients [55.3%]), lower respiratory tract infection (for 255 [10.6%]), and urinary tract infection (for 196 [8.1%]).

Overall, 176 of 4309 patients (4.1%) had blood cultures positive for pathogenic bacteria. The most common pathogens were *S.* Typhi (in 87 patients [49%]); *Staphylococcus aureus* (in 26 [15%])*, S.* Paratyphi (in 22 [13%]), and *Escherichia coli* (in 20 [11%]). Typhoidal *Salmonella* organisms were therefore detected in 2.5% of all patients and accounted for 62% of confirmed bacteremia cases. Dhulikhel Hospital had the highest proportion of patients presenting with *Salmonella* bacteremia (100 of 3529 [2.8%]), whereas Damauli Hospital had the lowest proportion (0 of 111).

**Figure 3.**
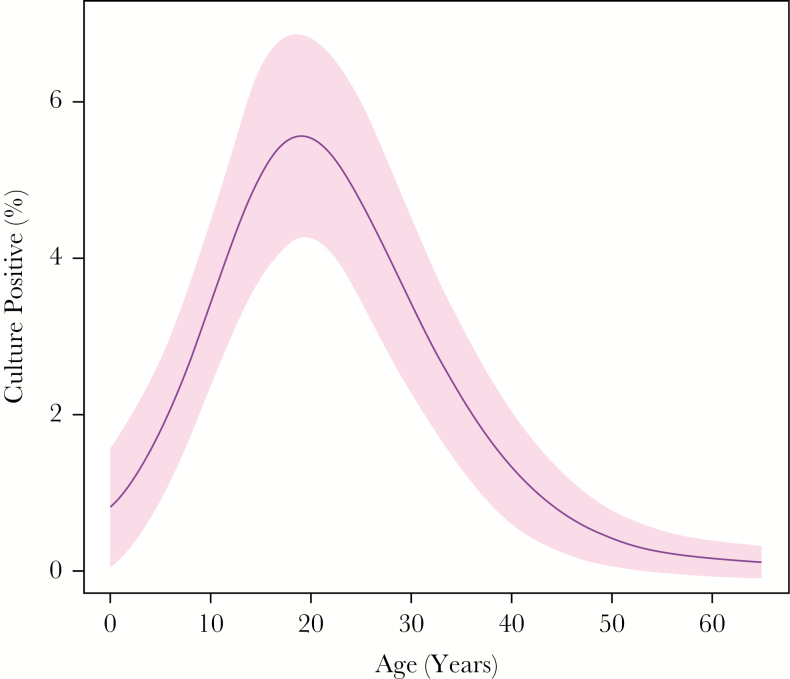
Proportion of patients with acute febrile illness who were blood-culture positive for typhoidal *Salmonella* organisms, by age. Curve and 95% confidence intervals were produced by a generalized additive model.

**Figure 4.**
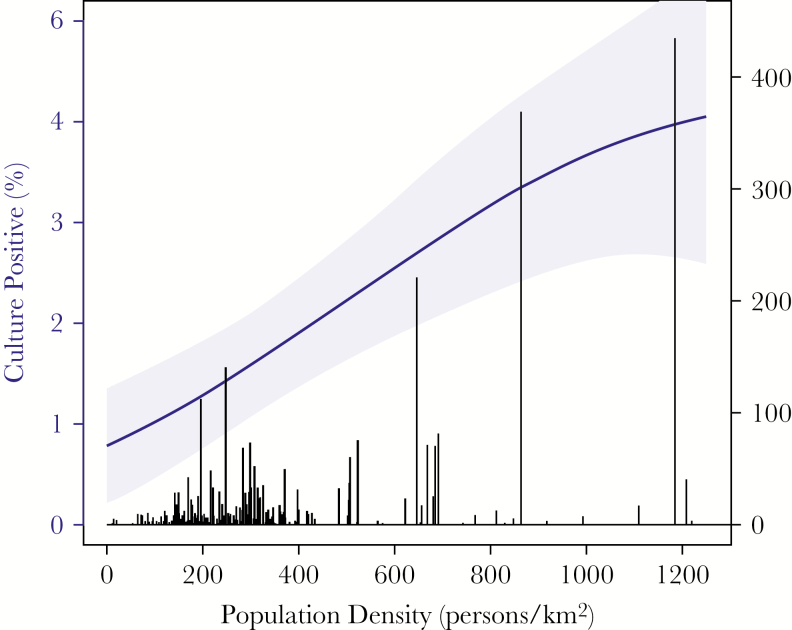
Culture positivity (blue line with shaded 95% confidence intervals) according to population density of the participant’s home locality. Number of study participants living in localities for a given population density is depicted by black histogram (right axis). Curve and 95% confidence intervals were produced by a generalized additive model.

Culture positivity for typhoidal *Salmonella* organisms was highest among participants aged 16–24 years (50 of 928 [5.4%]) and lowest among children aged <5 years (2 of 322 [0.6%]) and adults aged >50 years (1 of 1037 [0.1%]; Figure 3). Culture positivity was comparable among women (2.3%) and men (2.8%; *P* = .29; Table 1). Individuals who reported prior medical care during the febrile episode were more likely to be culture positive (3.0% vs 1.9%; *P* = .03). Additionally, there was a trend toward higher *Salmonella* culture positivity among participants who reported prior antibiotic use (3.0% vs 2.2%; *P* = .14). Patients with an empirical diagnosis of enteric fever were more likely to be culture positive for *Salmonella* organisms (55 of 1334 [4.1%]) than those with an alternative empirical diagnosis (54 of 2903 [1.9%]; *P* < .001). *Salmonella* culture positivity was higher among participants living in village development committees or municipalities with higher population density (Figure 4). Among those in localities with >1000 persons/km^2^, 4.9% were culture positive, compared with 1.1% among those living in localities with <200 persons/km^2^ (*P* < .001).

**Table 1.** Characteristics of Patients Presenting With Acute Febrile Illness With or Without Culture-Confirmed Enteric Fever

Characteristic	With Culture-Confirmed Enteric Fever	Without Culture-Confirmed Enteric Fever	*P*
Age, y			
<5	2/109 (2)	320/4200 (8)	<.001
5–15	29/109 (27)	780/4200 (19)	
16–24	50/109 (46)	868/4200 (21)	
25–49	27/109 (25)	1176/4200 (28)	
≥50	1/109 (1)	1036/4200 (25)	
Female sex	52/109 (48)	2238/4200 (53)	.291
Fever duration, d^a^	4 (4–7)	5 (3–7)	.733
Febrile at presentation	27/107 (25)	1109/4146 (27)	.811
Sought prior care during episode	72/108 (67)	2295/4122 (56)	.030
Reported antibiotic use within past 2 wk	45/96 (47)	1431/3675 (39)	.142
Clinical diagnosis			
Any, no.	109	3974	
Enteric fever	55/109 (50)	1279/3974 (32)	<.001
Lower respiratory tract infection	0/109 (0)	282/3974 (7)	.007
Upper respiratory tract infection	5/109 (5)	259/3974 (7)	.541
Urinary tract infection	3/109 (3)	207/3974 (5)	.355
Viral fever	3/109 (3)	126/3974 (3)	1.000
Tuberculosis	0/109	30/3974 (1)	.732
Admitted to hospital	17/107 (16)	958/4099 (23)	.090

Data are proportion (%) of patients or median value (interquartile range).

^a^Data are for 109 patients with and 4200 without culture-confirmed enteric fever.

All but 1 (101 of 102 [99%]) of the typhoidal *Salmonella* isolates tested were nonsusceptible to ciprofloxacin (intermediately resistant, 90 of 102; resistant, 11 of 102). However, the majority were susceptible to historically used first-line drugs, including trimethoprim-sulfamethoxazole (96 of 105 isolates [91%]), ampicillin (55 of 105 [52%]), and chloramphenicol (93 of 100 [93%]). Additionally, nearly all isolates were susceptible to ceftriaxone (99 of 103 [96%]), cefixime (33 of 37 [89%]), and azithromycin (104 of 106 [98%]). Individuals reporting prior antibiotic use were more likely to have isolates resistant to amoxicillin (40% vs 18%; *P* = .036), and there was a trend toward higher rates of resistance to trimethoprim-sulfamethoxazole (13% vs 2%; *P* = .053), chloramphenicol (12% vs 2%; *P* = .102), and ciprofloxacin (14% vs 7%; *P* = .310).

## DISCUSSION

Analyzing 20 years of data from health facilities throughout Nepal, we found very high and rising rates of enteric fever clinical diagnoses, particularly in rural areas, where blood culture data are sparse. We therefore undertook a prospective surveillance study of patients with acute febrile illness in health facilities serving peri-urban and rural communities and confirmed that typhoid was indeed the most common clinical diagnosis among patients presenting with a >72-hour history of fever. However, only 2.5% of all patients—and 4.1% of patients with an empirical diagnosis of enteric fever—were blood culture–positive for typhoidal *Salmonella* organisms. On the assumption that culture is 50% sensitive, these results imply that >90% of patients with a diagnosis of enteric fever actually had some other illness.

These results have several important implications for treatment of acute febrile illnesses and prevention of enteric fever in rural areas, where 81% of the Nepalese population resides [14]. First, many patients who presumably do not have enteric fever are receiving antibiotics targeted at typhoidal *Salmonella* organisms. For patients with other bacterial infections that appear common in Nepal, including scrub and murine typhus [17, 18], leptospirosis, and infection due to *S*. *aureus* (the second-most-commonly isolated pathogen in this study), the oral antibiotics commonly used as empirical therapy for enteric fever (levofloxacin, cefixime, and azithromycin) may be inadequate. Moreover, it is quite possible that many—if not the majority—of blood culture–negative patients have viral infections, for which antibiotics are ineffective and produce unnecessary toxicities and pressure for antimicrobial resistance. Second, characterizing the burden of enteric fever outside of Kathmandu is important to inform vaccine strategies, particularly in light of new conjugate vaccines, for which policy recommendations will be under consideration by the World Health Organization Strategic Advisory Group of Experts on Immunization in 2017 [9]. One key decision will be whether typhoid vaccination should be implemented nationally versus in geographically restricted, high-risk populations.

These results add to the literature suggesting that clinical diagnosis of enteric fever has a poor positive predictive value. Even when strict clinical criteria are derived and applied within the same setting [19–21], their positive likelihood ratios are insufficient for achieving high positive predictive values in settings where the prevalence is low. For example, based on the largest published clinical prediction study [21], applying the prediction rule with the highest specificity to the population of this study (with an up-adjusted 5% prevalence of typhoid among febrile patients) would yield a positive predictive value of 44%. This underscores the continued need for a better typhoid diagnostic [22].

In our study population, the highest number and proportion of individuals with culture-confirmed enteric fever were young adults (ie, individuals aged 16–24 years). Infection rates were low among young children and older adults. Although in high-incidence settings children appear to be at highest risk of typhoid, our findings are consistent with data from other low-burden settings, where the average age of infection increases and in some cases peaks in adolescents or young adults [11, 23, 24]. However, our data reflect cases and proportions, rather than incidence, and children may seek care for febrile illnesses at higher rates. Additionally, while sensitivity of small-volume blood cultures for children is thought to be comparable to that of larger-volume cultures for adults [25, 26], it is possible that lower culture sensitivity influenced the observed age distribution, as well.

Overall, we found that 2.5% of patients with acute febrile illness were culture positive for typhoidal *Salmonella* organisms. These culture-positivity proportions were lower than those reported from the Kathmandu valley. Karkey et al reported that 3898 of 54536 blood cultures (7.2%) were positive for typhoidal *Salmonella* species from 2005 to 2009 at Patan Hospital [11]. Among children aged 2 months to 14 years at that hospital, an outpatient study (conducted during 2006–2007) found that 12.6% of cultures were positive for typhoidal *Salmonella* organisms [27], while an inpatient study among children <13 years of age (conducted during 2005–2006) found that 2.9% of cultures were positive for typhoidal *Salmonella* species [28]. Bajracharya et al reported on 11 hospitals in Kathmandu, in which typhoidal *Salmonella* organisms were grown from 2933 of 27097 of cultures (10.8%) performed from 2008 to 2012 [13]. Recently, John et al reported on 37 studies from India, in which 9.7% of febrile patients (95% confidence interval, 5.7%–16.0%) were culture positive for *S.* Typhi, and 0.9% were positive for *S.* Paratyphi [29]. By contrast, culture-positivity rates among individuals with acute febrile illnesses at the 5 sites of the DOMI study were lower, ranging from 0.5% in Vietnam to 3.3% in North Jakarta [24]. We found that culture positivity was strongly associated with population density. Some recent studies, including those from Cambodia [30] and Ghana [31], have reported high rates of fever in rural areas, while others [32–35] have found much higher rates in urban as compared to rural settings within the same country. The risk of enteric fever may be highly local, likely related to characteristics of the water supply and opportunities for its contamination by sewage, which in some settings are enhanced by higher population densities. A better understanding of these drivers of typhoid risk could help inform targeted vaccine deployment.

As in other recent studies from South and Southeast Asia [8, 36, 37], we found high rates (99%) of fluoroquinolone nonsusceptibility, representing a sharp rise over those from the past several years at the study sites (unpublished data). In light of these findings and results of a recent randomized trial in Nepal that was stopped because of high failure rates with gatifloxacin [38], fluoroquinolones are no longer suitable for empirical therapy for enteric fever in this setting. Over the 3 years of the study, we noted an increase in prescription of azithromycin (from 43% to 52% of prescriptions; *P* < .001) and cefixime (from 32% to 56%; *P* < .001) for suspected enteric fever and a decrease in prescription of fluoroquinolones (from 13% to 3%; *P* = .005). Susceptibility to ceftriaxone, azithromycin, and cefixime remained high, although concerns remain about the use of cefixime in light of poor performance in trials in which it was compared to fluoroquinolones [39–41]. As has been observed recently in Kolkata and Lalitpur [42, 43], there has been a return of susceptibility to older first-line agents, including trimethoprim-sulfamethoxazole (91%) and chloramphenicol (93%). We do note, however, that antimicrobial resistance rates were higher among individuals who reported prior antibiotic use, suggesting that our hospital-based surveillance may be biased toward detecting resistance among patients who do not respond to outpatient antibiotic therapy.

The results of this study should be interpreted within the context of several limitations. First, blood culture, while highly specific, is an insensitive diagnostic for enteric fever. A recent systematic review estimated its sensitivity at 61% (95% confidence interval, 52%–70%) [44], while many experts have assumed even lower sensitivity [23] to account for the impact of antimicrobial use. A high proportion of patients in the study reported prior antibiotic exposure, which may have diminished culture sensitivity. Interestingly, we found that individuals who reported prior antibiotic use during the febrile episode had a trend toward a higher frequency of culture positivity (3.0% vs 2.2%; *P* = .14). This was presumably because persistent symptoms despite prior antibiotic therapy may be an indicator of more-severe disease, consistent with enteric fever. However, self-report has been found to be an unreliable marker of antibiotic exposure in previous studies in Nepal and Laos [17, 45]. Future work relating specific antibiotic exposures and culture yield could improve estimation of disease burden. We measured the proportion of patients who were culture positive for typhoidal *Salmonella* species among those with 3 days of fever; however, these data do not enable direct conversion to population incidence rates, owing to variable care-seeking behaviors. As part of the Surveillance for Enteric Fever in Asia Project, studies are now underway to measure healthcare utilization for acute febrile illnesses in the facility catchment areas, which will be used to estimate incidence [3, 46]. Our data included 4 health facilities and participants from 14 of Nepal’s 75 districts; however, these may not be generalizable throughout rural Nepal. Finally, key remaining questions, unanswered by this study, are the etiologies for the other 95% of acute febrile illnesses. While studies have demonstrated the importance of rickettsia and leptospirosis in the Kathmandu valley [17, 18, 47], there are no published data on the causes of febrile illness in the remainder of the country. Future work will include molecular and serological assays to better characterize the range of etiologies of fever in the rural and peri-urban populations.

Enteric fever remains an important cause of morbidity and mortality globally; however, regional burden estimates for South Asia have been derived exclusively from urban settings [5]. There are few published data from rural South Asia and, prior to this study, none from rural Nepal. We found very high rates of enteric fever clinical diagnosis in rural areas; however, the proportion of culture-confirmed disease was very low and strongly related to population density. These findings underscore the need to address current practices of widespread antibiotic prescription for presumptive enteric fever in rural areas and to generate higher resolution geographical data on typhoid burden to inform future vaccine implementation strategies.

## Notes

***Disclaimer.*** The funders had no role in study design, data collection and analysis, decision to publish, or preparation of the manuscript.

***Financial support.*** This work was supported by the Burroughs Wellcome Fund/American Society of Tropical Medicine and Hygiene (fellowship to J. R. A.), the Rosenkranz Prize for Healthcare Research, the Charles A. King Trust Fellowship, and the National Institute of Allergy and Infectious Diseases (AI100023). This article is part of the supplement “Surveillance of Enteric Fever in Asia Project,” sponsored by the Sabin Vaccine Institute. This publication was made possible by a grant from the Bill and Melinda Gates Foundation (OPP11130007).

***Supplement sponsorship.*** This article is part of the supplement “Surveillance for Enteric Fever in Asia Project,” sponsored by the Sabin Vaccine Institute.

***Potential conflicts of interest.*** All authors: No reported conflicts of interset. All authors have submitted the ICMJE Form for Disclosure of Potential Conflicts of Interest. Conflicts that the editors consider relevant to the content of the manuscript have been disclosed.
